# Association of Genome-Wide Association Study (GWAS) Identified SNPs and Risk of Breast Cancer in an Indian Population

**DOI:** 10.1038/srep40963

**Published:** 2017-01-18

**Authors:** Rajini Nagrani, Sharayu Mhatre, Preetha Rajaraman, Nilanjan Chatterjee, Mohammad R. Akbari, Paolo Boffetta, Paul Brennan, Rajendra Badwe, Sudeep Gupta, Rajesh Dikshit

**Affiliations:** 1 Centre for Cancer Epidemiology, Tata Memorial Centre, Mumbai, India; 2 Division of Cancer Epidemiology & Genetics, National Cancer Institute, Bethesda, USA; 3 Department of Biostatistics, Bloomberg School of Public Health, Johns Hopkins University, USA; 4 Department of Oncology, School of Medicine, Johns Hopkins University, USA; 5 Women’s College Research Institute, Women’s College Hospital, Toronto, ON, Canada; 6 Dalla Lana School of Public Health, University of Toronto, Toronto, ON, Canada; 7 Institute For Translational Epidemiology, Mount Sinai Hospital, One Gustave L.Levy Place New York, NY, USA; 8 Genetic Epidemiology Group, International Agency for Research on Cancer, 150 Cours Albert Thomas, 69372 Lyon CEDEX, France; 9 Department of Surgical Oncology, Tata Memorial Hospital, Mumbai, India

## Abstract

To date, no studies have investigated the association of the GWAS-identified SNPs with BC risk in Indian population. We investigated the association of 30 previously reported and replicated BC susceptibility SNPs in 1,204 cases and 1,212 controls from a hospital based case-control study conducted at the Tata Memorial Hospital, Mumbai. As a measure of total susceptibility burden, the polygenic risk score (PRS) for each individual was defined by the weighted sum of genotypes from 21 independent SNPs with weights derived from previously published estimates of association odds-ratios. Logistic regression models were used to assess risk associated with individual SNPs and overall PRS, and stratified by menopausal and receptor status. A total of 11 SNPs from eight genomic regions (*FGFR2, 9q31.2, MAP3K, CCND1, ZM1Z1, RAD51L11, ESR1* and *UST*) showed statistically significant (p-value ≤ 0.05) evidence of association, either overall or when stratified by menopausal status or hormone receptor status. BC SNPs previously identified in Caucasian population showed evidence of replication in the Indian population mainly with respect to risk of postmenopausal and hormone receptor positive BC.

Breast cancer (BC) is a complex disease strongly influenced by genetic, environmental and lifestyle factors. Under the assumption that common disorders in humans are partly due to common low penetrant polymorphisms, a large number of genome wide association studies (GWAS) have been conducted during the last decade, primarily in Caucasian, European, Japanese and Chinese populations^1^,^2^,^3^. These studies along with projects such as HapMap and 1000G indicate that the prevalence of single nucleotide polymorphisms (SNPs) can vary in different populations, and in some cases, the risk estimates associated with BC also differ^4^,^5^,^6^,^7^.

BC rates have been increasing in India for the last decade. However, there are very few large-scale population based studies to identify risk factors related to lifestyle and/or genetics. Given that a high percentage of BC in India occurs in young and premenopausal women^8^, with a large proportion being triple negative tumours that typically have poorer prognosis^9^, it is important to understand the aetiology of BC in this population. We conducted a large scale case-control study at Tata Memorial Hospital (TMH), Mumbai, India, to identify lifestyle and genetic risk factors for BC. In this analysis, we focus on examining risk in established SNP loci for BC identified by GWAS studies in Caucasian and East Asian populations. To the best of our knowledge this is the first study in an Indian population to examine a large number of GWAS-identified BC risk loci in an adequately-powered population-based study.

## Methods

We conducted a hospital based case-control study at TMH, Mumbai during the period of January 2009 to September 2013. Enrolled cases were females with primary BC seen at TMH, aged 20–69 years with a date of diagnosis not more than 6 months from date of interview. All BC cases enrolled in the study were histologically confirmed.

Female visitors aged 20–69 years, with no history of cancer, and who were accompanying cancer patients of any primary site, were eligible for inclusion as controls. Controls that were unrelated to BC patients were genotyped and included in the analysis. Controls were frequency-matched to cases by age and region of residence at enrolment. The study has been approved by TMH Institutional Review Board. Written informed consent was obtained from all study participants before enrolling them in the study. All methods were performed in accordance with the relevant guidelines and regulations.

Information collected on all subjects included data on residential status, reproductive history and anthropometric measurements. Women whose menstrual period had either stopped naturally, or due to oophorectomy, hysterectomy or any other reason for 12 months or more before the date of enrolment were classified as postmenopausal. The rest were treated as premenopausal. Estrogen receptor (ER), progesterone receptor (PR) and human epidermal growth factor receptor 2 (HER2) status for cases were obtained from hospital pathology records.

Apart from questionnaire data and anthropometric measurements a 10 ml blood sample was collected from each study participant and plasma and buffy coat were separated. Buffy coat samples were available for 1,214 cases (74.1% of all cases) and 1,293 controls (85.3% of all controls).

### DNA Preparation and Assay Performance

Genomic DNA was extracted from buffy coat using the Qiagen QiAamp Blood DNA MidiKit and Macherey Nagel Nucleomag Blood kit. The concentration of each DNA sample was determined by Quant-iT PicoGreen assay. A total of 250 ng DNA was applied to SNP typing using Illumina’s GoldenGate Genotyping Custom SNP Panel assay (Illumina Inc., San Diego, CA)^10^. Illumina Golden Gate assay is reported to be highly accurate in humans with error rates in the order of 0.3–0.4%^11^. Genotyping was performed on 1,204 cases and 1,212 controls on 384 custom-selected SNPs. Plates were prepared containing randomly mixed cases and controls. Intraplate and interplate replicates (7% approx.) were included on all plates and in all batches.

### Design of Custom SNP Panel

A customized panel of 384 SNPs was designed by including GWAS-identified BC risk loci, BC SNPs identified from candidate gene studies, SNPs previously reported to be associated with obesity related traits and other SNPs in obesity genes. This paper focuses on a subset of 31 BC GWAS SNPs identified in Caucasian and East Asian populations using the Human Genome Epidemiology (HuGE) Navigator and the National Institute of Health (NIH) GWAS Catalog^12^,^13^. We have also included 45 BC SNPs from candidate gene studies. Only 30 BC GWAS SNPs and 42 BC candidate SNPs were used for final analysis after quality assessment. For BC GWAS SNPs, only SNPs with p value < 5 × 10^−8^ were included in the analysis. Duplicate SNPs between the HuGE Navigator and NIH GWAS Catalog were removed. There were no overlapping SNPs between GWAS and candidate gene studies. The panel was designed in March 2011 and therefore SNPs identified after this time were not included.

Since results from candidate gene SNP studies have had a poor record of replication^14^, we have presented the results in Supplementary Table S3 and have not evaluated them in the main tables.

### Quality Assessment

The reproducibility rate of replicate samples (n = 160) for all assays was >98%. Examination of negative controls indicated no inter-sample contamination. A designability rank score (0–1.0) was calculated for each SNP by Illumina for conversion of SNP into a successful GoldenGate Assay. All SNPs had a score of 1.0, indicating a high success rate. Following completion of the assay, data were cleaned using the Illumina Genome Studio software version 1.9.4. Automatic allele calling was performed using a GenCall (GC) threshold of 0.25. The software assigned three clusters on a graph based on the fluorescence obtained. Seventeen samples had a call rate <90% that were excluded and a total of 2,399 (1,194 cases and 1,205 control) samples were included in the final analysis. One SNP with MAF <1% and 3 SNPs with diffused clusters in our study population were excluded, yielding a list of 30 GWAS SNPs and 42 candidate SNPs (i.e. total 72 SNPs across 41 genes) for final analysis. All SNPs had call frequency above 95%. No deviation from hardy-weinberg equilibrium (HWE) (*P* < 0.001) was observed using the chi-square test, and all SNPs had Gen-train score value of 0.4 and above. All quality control dashboards provided in Genome Studio Software showed that the quality of the assays were satisfactory including allele specific extension, PCR uniformity, extension gap, first and second hybridization.

Out of 1,194 cases and 1,205 controls, information on menopausal status could be obtained on 1,193 cases (607 premenopausal and 586 postmenopausal) and 1,191 (650 premenopausal and 541 postmenopausal) controls respectively. When we stratified cases by hormone receptor status, there were 408 estrogen receptor positive (ER+)/progesterone receptor positive (PR+), 529 estrogen receptor negative (ER−)/progesterone receptor negative (PR−) irrespective of their HER2 status and 340 triple negative breast cancer (TNBC) cases.

### Statistical Analysis

Unconditional logistic regression was used to estimate adjusted odds ratios (OR) and corresponding 95% confidence intervals (CI) between genotype and BC case-control status. The model was adjusted for age (continuous variable) and region of residence (North, South, East, West and Central India). An additive model of inheritance (continuous effect of increasing number of variant alleles - 0 versus 1 versus 2) was assumed and the genotypes were coded as 0 = wild type, 1 = heterozygous and 2 = homozygous variant. Further analyses were performed by menopausal and hormone receptor status. ORs for all SNPs were reported with respect to the risk allele as identified in previous GWAS. All statistical tests were two-sided, and a P value equal to or less than 0.05 was considered statistically significant.

To investigate the association between BC risk and total susceptibility burden defined by the combination of the SNPs, a polygenic risk score (PRS) was derived for each individual using the formula: PRS = β_1_x_1_ + β_2_x_2_ + … + β_n_x_n_. For any given SNP n, β_n_ is the log-odds-ratio associated with risk allele reported in the literature from prior GWAS conducted in Caucasian population^2^ and x_n_ is the number of risk alleles carried by an individual in our study. Only independent SNPs were included in the PRS analysis. If there were multiple SNPs in LD (r^2 > 0.2) from one region, the SNP with strongest association signal reported in previous GWAS was picked for our analysis, resulting in a total of 21 independent SNPs. Logistic regression models were used to estimate the odds ratios for BC by percentile of the PRS, with 25^th^ percentile as the reference. Based on previously reported ORs of all the SNPs, their allele frequencies in the Indian population, and the sample, we estimated power of replication of each SNP in the current study.

The probability of observing a larger number of significant associations (*P* ≤ 0.05) than would be expected by chance is a function of the binomial distribution^15^. We conducted a global test for the hypothesis to evaluate whether the number of significant associations with BC (*P* ≤ 0.05) was greater than expected for the number of loci tested. All analyses were performed using the statistical software Stata version 12.0^16^.

## Results

A total of 1,194 cases and 1,205 controls were included in the final analysis. Table 1 describes the distribution of cases and controls with respect to age, education, region of residence at enrolment, menopausal status, and family history of breast, ovary or endometrial cancer. The risk of TNBC increases two-fold in females with family history of breast, ovary or endometrial cancer (OR = 2.00; 95%CI = 1.01–3.97) (data not shown). Direction of effect were similar to previously reported GWAS for 22 of the 30 SNPs, but different for SNPs rs10069690 (*TERT*), rs13387042 (*TNP1*), rs1562430 (*FAM84B*), rs2180341 (*RNF146*), rs2981575 (*FGFR2*), rs3757318 (*C6orf97*), rs6504950 (*STXBP4*), rs999737 (*RAD51L1*) (Table 2). For the risk of overall BC, we confirm previously-reported associations between 5 GWAS-identified BC susceptibility SNPs, with an exception of rs2981575 which was associated with BC risk in our population but with reverse direction of effect (Table 2). Overall, out of the 30 GWAS BC SNPs analysed 22 SNPs showed effects in the same direction as that reported in previous GWAS. Applying the binomial test for enrichment indicated that the pattern was unlikely to be due to chance (p-value = 0.016). When cases were stratified by menopausal status, a number of BC GWAS-identified SNPs appeared to show stronger association in postmenopausal versus premenopausal women (Table 3). In particular, 7 SNPs achieved statistical significance (*P* ≤ 0.05) for association with postmenopausal BCs: *FGFR2* (rs1219648, rs2981575, rs2981579 and rs2981582), *MAP3K1* (rs889312), *ESR1* (rs2046210) and 9q31.2 (rs865686) whereas only one SNP *RAD51L1* (rs999737) showed association with premenopausal BCs. Details of all SNPs stratified on menopausal status are presented in Supplementary Table S1. Analyses performed on ER/PR status showed a total of 8 SNPs achieved statistical significance for association with ER+/PR+ BC. In addition, the SNP rs2046210 in *ESR1* and rs9485372 (*UST*) showed statistically significant increased risk for BCs for ER−/PR− and TNBC but not for ER+/PR+ (Table 4). The minor alleles of rs1219648, rs2981579, rs2981582 (*FGFR2*), rs889312 (*MAP3K1*), rs614367 (*CCND1*) and rs704010 (*ZMIZ1*) increased the risk. The alleles T and C of rs2981575 (*FGFR2*) and rs999737 (*RAD51L1*) respectively decreased the risk of hormone receptor positive BC (Table 4). Details of all SNPs analysed by hormone receptor status are presented in Supplementary Table S2.

The distribution of the PRS was shifted upwards in cases when compared to controls. Among all the different outcomes considered, PRS showed strongest association with the risk of BC among postmenopausal cases. In particular, postmenopausal women in the highest quartile of the PRS score had an 83% increased BC risk when compared to women in the lowest quartile. Results were similar when study participants with family history of breast, ovary or endometrial cancer were excluded from analysis (Table 5).

We also attempted to replicate associations for 42 BC SNPs which were previously identified in candidate studies and observed significant association in 3 regions viz. rs2420946 (*FGFR2*), rs3218408 (*XRCC2*), rs1641535 and rs1641536 (*ATP1B2*) (Supplementary Table S3).

## Discussion

We used 1,194 cases and 1,205 controls from a hospital based case-control study in India to report evidence of replication for susceptibility BC SNPs that have been previously identified primarily through GWAS conducted in Caucasian population. To our knowledge this is the first study to evaluate risk of BC and GWAS-identified SNPs in the Indian population. The study population is unique in that it is an unscreened population with no use of hormone replacement therapy and 52% premenopausal women. The average tumour size in our study cases was well over 2 cm^17^ and cases were not diagnosed mammographically.

Out of 30 GWAS-identified SNPs analysed, 11 SNPs from eight genomic regions (*FGFR2, 9q31.2, MAP3K1, CCND1, ZM1Z1, RAD51L11, ESR1* and *UST*) showed statistically significant (*P* ≤ 0.05) evidence of association either overall or when stratified by menopausal status or when analysed separately by hormone receptor status BCs. The direction of effect was the same as the previously reported GWAS results for 22 of the 30 SNPs.

We also attempted to replicate associations for 42 SNPs reported to be associated with BC in candidate gene studies. We observed statistical significance in 4 SNPs (9.5%) indicating that association observed in candidate gene studies are more prone to false positive results.

Our current data support the conclusions of previous GWAS studies^18^,^19^ that *FGFR2* (a tumour suppressor gene) polymorphisms (rs2981582 and rs2981575), first identified as susceptibility loci for BC in Caucasian population^20^,^21^, are associated with overall BC. The risk allele frequencies of *FGFR2* SNPs were similar to those reported in previous GWAS, with the exception of rs2981575, which was much common in our population. rs865686 in *9q31.2* was significantly associated with BC, further the rare allele (G) frequency of rs865686 (MAF = 0.14) obtained in our study was comparable to that reported in a previous study in Asians (MAF = 0.09)^22^.

Consistent with results from Caucasian and East Asian populations, we found that both rs889312 (*MAP3K1*) and rs2046210 (close to *ESR1*) were associated with increased risk of overall BC in our Indian population. rs889312 lies in an LD block of approximately 280 kb which includes the *MAP3K1* gene^23^. The *MAP3K1* gene encodes a 196-kDa serine/threonine protein kinase that activates the extracellular signal regulated kinase (ERK), c-Jun NH2-terminal kinase (JNK) and nuclear factor-kB (NF-kB) pathways^24^. Downstream signal transduction genes regulate cell survival, differentiation, proliferation and apoptosis, and appear to be involved in tumour development and tumour progression^25^,^26^,^27^.

The SNP rs2046210 is located 180 kb upstream of the transcription initiation site of the first coding exon of the *ESR1* gene. Considering the relative vicinity of rs2046210 to the *ESR1* gene, it has been speculated that either the SNP itself, or causal variants in LD with it, might alter *ESR1* gene expression, thus affecting susceptibility to BC. Functional genomic analyses and *in vitro* functional experiments conducted by Cai *et al*.^28^ provided no support for the potential involvement of the polymorphism itself in the regulation of *ESR1*. The function of this SNP therefore is still unclear; future fine-mapping of the BC susceptibility loci tagged by rs2046210 is warranted and the underlying biological mechanism of this polymorphism needs further investigation.

Results of our hormone receptor analyses successfully replicated previous GWAS reported loci for hormone receptor positive BCs in *FGFR2* (rs1219648, rs2981575, rs2981579 and rs2981582)^29^; *MAP3K1* (rs889312)^30^,^31^, *CCND1* (rs614367) *ZMIZ1* (rs704010)^3^, although the inverse direction of effect as compared to the previous GWAS report for *FGFR2* (rs2981575) and *RAD51L1* (rs999737) was unexpected. Our observed association of increased risk with respect to rs2046210 (*ESR1*) was consistent with previous studies suggesting that rs2046210 tended to increase BC risk in ER− tumour by a greater magnitude as compared to ER+ tumour^32^,^33^,^34^. Consistent with the literature, we also found SNP rs9485372 (*UST*) to be associated more with ER−/PR− and TNBC^1^.

Our analyses stratified on menopausal status showed 7 SNPs to be associated with postmenopausal BC, as opposed to only 1 SNP associated with premenopausal BC. SNPs in *FGFR2* (rs2981582, rs2981575, rs1219648 and rs2981579), *MAP3K1* (rs889312) and the *9q31.2* region (rs865686) were associated with BCs in postmenopausal women. Large scale GWAS studies have previously reported that the association of rs865686 is stronger in postmenopausal women^2^,^22^,^35^. Our observed associations of rs2046210 and BC were also consistent with prior literature suggesting that risk in postmenopausal BC cases was greater than risk in premenopausal BC cases^4^.

We observed evidence of replication of association for previously reported GWAS BC SNPs mostly among postmenopausal or ER+/PR+ BC patients. This is not surprising given that these SNPs were identified mainly in American/European populations comprised largely of postmenopausal/older women^21^,^36^,^37^. Among ER+/PR+ SNPs, most of SNPs (80%) were significant for postmenopausal women (data not shown). None of 19 SNPs which could not be replicated in present study (except rs2180341) had statistical power of 80% or more for replication (Supplementary Table S4), suggesting that larger sample sizes may be needed in order to detect an association. Nonetheless, the consistency analysis showed that the similarity in direction of effect for these 18 non-replicated SNPs was more than chance (*P* = 0.016). On the other hand, the null observation in our study for rs2180341 (an association initially observed in Ashkenazi Jewish GWAS population) even with 98% power for replication, is more likely to reflect a true difference in association across different ethnicities.

Our results of the 21 SNP PRS (including only the strongest SNP from LD >0.2 SNP groups) showed that postmenopausal women in the highest quartile of PRS had an OR of 1.83 (95% CI = 1.28–2.60) when compared to women in lowest quartile. An increase in risk of ER+/PR+ BCs was also observed, having OR = 1.36 (95% CI = 1.04–1.79) with per unit increase in PRS.

In conclusion, our study provides early evidence that the genetic architecture of postmenopausal and/or hormone receptor positive BC in the Indian population may be similar to that of Caucasian populations. More population-based studies in the Indian population are needed in order to identify additional BC susceptibility SNPs, especially for hormone receptor negative BCs.

## Additional Information

**How to cite this article**: Nagrani, R. *et al*. Association of Genome-Wide Association Study (GWAS) Identified SNPs and Risk of Breast Cancer in an Indian Population. *Sci. Rep.* **7**, 40963; doi: 10.1038/srep40963 (2017).

**Publisher's note:** Springer Nature remains neutral with regard to jurisdictional claims in published maps and institutional affiliations.

## Supplementary Material

Supplementary Dataset 1

## Figures and Tables

**Table 1:** Summary Characteristics of Study Population.

Parameters		% Cases (N = 1194)	% Controls (N = 1205)
Age at enrolment (years)	20–294	3.0	2.5
	30–39	22.7	23.1
	40–49	36.3	36.7
	50–59	26.9	27.6
	60–69	11.1	10.2
	Mean(±SD)	46.0 (±9.6)	45.6 (±9.6)
	Missing	0	0
Region of residence at enrolment	North	21.9	21.8
	West	49.3	49.7
	Central	5.4	5.3
	East	22.2	21.9
	South	1.2	1.3
	Missing	0	0
Education	No formal schooling	21.0	18.7
	<5 yrs of schooling	6.5	6.4
	5–8 yrs of schooling	23.8	24.8
	High School	27.4	29.9
	College graduation & more	21.2	20.1
	Missing	0.2	0.1
Family History of Breast, Ovary or Endometrial Cancer	No	96.1	97.4
	Yes	3.7	2.2
	Missing	0.3	0.4
Menopausal Status	Premenopausal	50.8	54.2
	Postmenopausal	49.1	45.1
	Missing	0.1	0.8

**Table 2:** Association and comparison of allele frequencies for GWAS identified BC SNPs with Indian population.

Locus	SNP ID	Chr	Gene Symbol	Ethnicity	Details of Previous GWAS				Details of Present Case-Control Study (Cases = 1,194 Control = 1,205)				PRS Analysis
					PMID	Risk Allele (RAF)	OR^b^ (95%CI)	p- value	EAF^a^	Case/Control	OR^b^ (95% CI)	p-value	
1	rs11249433	1	LOC647121	European	23535729	G (0.40)	1.09 (1.07–1.11)	2.0 × 10^−26^	0.17	1174/1190	1.04 (0.89–1.21)	0.598	Yes
2	rs13387042	2	TNP1	European	23535729	A (0.51)	1.14 (1.11–1.16)	2.0 × 10^−57^	0.51	1188/1196	0.93 (0.83–1.04)	0.221	Yes
3	rs4973768	3	SLC4A7	European	23535729	T (0.47)	1.10 (1.08–1.12)	2.0 × 10^−30^	0.45	1189/1198	1.03 (0.92–1.16)	0.547	Yes
4	rs10069690	5	TERT	European	23535733	T (0.32)	1.15 (1.11–1.20)	5.0 × 10^−12^	0.29	1187/1197	0.95 (0.84–1.08)	0.489	Yes
5	rs10941679	5	FGF10	European	23535729	G (0.25)	1.13 (1.10–1.15)	2.0 × 10^−37^	0.39	1190/1200	1.00 (0.89–1.12)	0.958	Yes
5	rs4415084	5	FGF10	European	21263130	T (0.42)	1.17 (1.11–1.22)	8.0 × 10^−11^	0.52	1184/1197	1.03 (0.91–1.16)	0.586	
6	rs889312	5	MAP3K1	European	23535729	C (0.28)	1.12 (1.10–1.15)	3.0 × 10^−36^	0.39	**1178/1178**	**1.16 (1.03–1.30)**	**0.009**	Yes
7	rs2046210	6	ESR1	European	23535733	A (0.42)	1.15 (1.11–1.19)	5.0 × 10^−16^	0.35	**1189/1198**	**1.14 (1.01–1.29)**	**0.027**	Yes
				Chinese	19219042	A (0.37)	1.29 (1.21–1.37)	2.0 × 10^−15^					
8	rs2180341	6	RNF146	Ashkenazi Jews	18326623	G (0.21)	1.41 (1.25–1.59)	3.0 × 10^−8^	0.41	1182/1192	0.95 (0.84–1.06)	0.408	Yes
9	rs3757318	6	C6orf97	European	23535729	A (0.07)	1.16 (1.12–1.21)	2.0 × 10^−21^	0.07	1193/1202	0.99 (0.79–1.24)	0.951	Yes
10	rs9485372	6	UST	East Asian	22383897	G (0.55)	1.11 (1.09–1.15)	3.8 × 10^−12^	0.79	1183/1199	1.09 (0.94–1.25)	0.228	
11	rs©13281615	8	FAM84B	European	23535729	G (0.41)	1.09 (1.07–1.12)	1.0 × 10^−27^	0.5	1183/1189	1.06 (0.94–1.19)	0.299	Yes
11	rs1562430	8	FAM84B	European	21908515	A (0.60)	1.16 (1.11–1.22)	3.1 × 10^−11^	0.77	1185/1204	0.99 (0.86–1.13)	0.926	
12	rs1011970	9	CDKN2BAS	European	20453838	T (0.17)	1.09 (1.04–1.14)	3.0 × 10^−8^	0.26	1187/1201	1.13 (0.99–1.29)	0.057	Yes
13	rs865686	9	9q31.2	European	23535729	T (0.62)	1.12 (1.11–1.14)	9.5 × 10^−35^	0.86	**1191/1199**	**1.25 (1.06–1.48)**	**0.008**	Yes
14	rs10822013	10	ZNF365	East Asian	21908515	T (0.47)	1.12 (1.06–1.18)	6.0 × 10^−9^	0.5	1188/1193	1.01 (0.89–1.13)	0.852	
14	rs10995190	10	ZNF365	European	23535729	G (0.84)	1.16 (1.14–1.19)	1.0 × 10^−36^	0.92	1194/1205	1.12 (0.90–1.39)	0.278	Yes
15	rs1219648	10	FGFR2	British	21263130	G (0.42)	1.31(1.25–1.37)	1.0 × 10^−30^	0.37	1188/1196	1.12 (0.99–1.26)	0.054	
15	rs2981575	10	FGFR2	European	21060860	T (0.42)	1.28 (1.18–1.39)	1.0 × 10^−8^	0.62	**1187/1193**	**0.88 (0.78–0.99)**	**0.042**	
15	rs2981579	10	FGFR2	European	23535729	A (0.40)	1.27 (1.24–1.29)	2.0 × 10^−170^	0.4	1192/1205	1.10 (0.98–1.24)	0.084	Yes
15	rs2981582	10	FGFR2	European	17529967	A (0.38)	1.26 (1.23–1.30)	2.0 × 10^−76^	0.33	**1194/1204**	**1.15 (1.02–1.31)**	**0.019**	
16	rs704010	10	ZMIZ1	European	23535729	T (0.38)	1.08 (1.06–1.10)	7.0 × 10^−22^	0.29	1192/1198	1.06 (0.93–1.21)	0.327	Yes
17	rs3817198	11	LSP1	European	23535729	C (0.31)	1.07 (1.05–1.09)	2.0 × 10^−11^	0.36	1189/1194	1.05 (0.93–1.18)	0.394	Yes
18	rs614367	11	CCND1	European	23535729	T (0.15)	1.21 (1.18–1.24)	2.0 × 10^−63^	0.15	1183/1189	1.14 (0.97–1.33)	0.095	Yes
19	rs999737	14	RAD51L1	European	23535729	C (0.77)	1.09 (1.06–1.11)	3.0 × 10^−19^	0.88	1194/1204	0.92 (0.78–1.10)	0.412	Yes
20	rs3112612	16	TOX3	European	21263130	T (0.43)	1.15 (1.10–1.21)	4.0 × 10^−10^	0.46	1187/1197	1.03 (0.92–1.15)	0.559	
20	rs3803662	16	TOX3	Japanese	24143190	T (0.52)	1.21 (1.14–1.28)	3.0 × 10^−11^	0.28	1190/1195	1.07 (0.95–1.22)	0.235	Yes
				European	23535729	A (0.26)	1.24 (1.21–1.27)	2.0 × 10^−114^					
20	rs4784227	16	TOX3	East Asian	22383897	T (0.24)	1.24 (1.20–1.29)	1.0 × 10^−28^	0.22	1189/1201	1.10 (0.96–1.26)	0.162	
21	rs6504950	17	STXBP4	European	23535729	G (0.72)	1.06 (1.04–1.09)	2.3 × 10^−13^	0.84	1190/1203	0.91 (0.78–1.06)	0.264	Yes
22	rs8170	19	C19orf62	European	23535733	A (0.19)	1.15 (1.11–1.20)	9.0 × 10^−13^	0.1	1190/1204	1.05 (0.87–1.27)	0.579	Yes

Abbreviations: C19orf62, Chromosome 19 Open Reading Frame 62; C6orf97, Chromosome 6 Open Reading Frame 97; CCND1, Cyclin D1; CDKN2BAS, Cyclin-Dependent Kinase Inhibitor 2B, Antisense; Chr, Chromosome; CI, Confidence Interval; EAF, Effect Allele Frequency; ESR1, Estrogen receptor 1; FAM48B, Family With Sequence Similarity 84, Member B; FGF10, Fibroblast Growth Factor 10; FGFR2, Fibroblast Growth Factor Receptor 2; GWAS, Genome Wide Association Studies; HCN1, Hyperpolarization-Activated Cyclic Nucleotide-Gated Potassium Channel 1; LSP1, Lymphocyte-Specific Protein; MAP3K1, Mitogen-Activated Kinase Kinase Kinase 1; OR, Odds Ratio; PRS, Polygenic risk ScoreRAD51L1, RAD51 Paralog B; RNF146, Ring Finger Protein 146; SNP, Single Nucleotide Polymorphism; STXBP4, Syntaxin-Binding Protein 4; TERT, Telomerase Reverse Transcriptase; TNP1, Transition Protein 1; TOX3, Tox High Mobility Group Box Family Member 3; UST, Uronyl 2-Sulfotransferase;, ZMIZ1, Zinc Finger Miz-Domain Containing 1; ZNF365, Zinc Finger Protein 365.

^a^Effect Allele Frequency in controls with respect to risk alleles identified in previous GWAS.

^b^Adjusted for age and region of residence.

Allelic model of inheritance was fitted in previous GWAS with an exception of rs9485372 (GG v/s AA).

Total number per SNP may vary because of missing values.

Significant association in the present study are shown in bold.

SNPs in FGFR2, ZNF365, TOX3, FAM84B, FGF10 are in LD >0.20.

**Table 3:** Association of SNPs identified in BC GWAS and risk of BC in present study stratified on Menopausal Status.

SNP ID	Gene Symbol	Effect Allele	EAF^a^	Postmenopausal (Cases = 586 Control = 541)			Premenopausal (Cases = 607 Control = 650)		
				Case/Control	OR^b^ (95% CI)	p-value	Case/Control	OR^b^ (95% CI)	p-value
rs1219648	FGFR2	G	0.37	**584/537**	**1.22 (1.02–1.45)**	**0.026**	603/645	1.04 (0.88–1.22)	0.632
rs2046210	ESR1	T	0.35	**584/539**	**1.22 (1.01–1.46)**	**0.031**	604/645	1.09 (0.93–1.28)	0.263
rs2981575	FGFR2	T	0.62	**582/538**	**0.81 (0.68–0.96)**	**0.019**	604/641	0.95 (0.81–1.12)	0.585
rs2981579	FGFR2	T	0.40	**585/541**	**1.19 (1.008–1.42)**	**0.040**	606/650	1.03 (0.88–1.21)	0.680
rs2981582	FGFR2	T	0.33	**586/541**	**1.29 (1.07–1.55)**	**0.006**	607/649	1.05 (0.88–1.24)	0.557
rs865686	9q31.2	T	0.86	**584/539**	**1.30 (1.02–1.65)**	**0.030**	606/646	1.21 (0.96–1.54)	0.102
rs889312	MAP3K1	C	0.39	**578/529**	**1.24 (1.04–1.47)**	**0.013**	599/635	1.10 (0.94–1.30)	0.210
rs999737	RAD51L1	C	0.88	586/540	1.13 (0.88–1.46)	0.316	**607/650**	**0.78 (0.61–0.99)**	**0.045**

Abbreviations: Chr, Chromosome; CI, Confidence interval; EAF, Effect Allele Frequency; ESR1, Estrogen receptor 1; FGFR2, Fibroblast Growth Factor Receptor 2; GWAS, Genome Wide Association Studies; MAP3K1, Mitogen-Activated Kinase Kinase Kinase 1; OR, Odds Ratio; RAD51L1, RAD51 Paralog B; SNP, Single Nucleotide Polymorphism.

^a^Effect Allele Frequency in controls.

^b^Adjusted for age and region of residence.

Total number per SNP may vary because of missing values.

Significant associations are shown in bold.

Significant association in the present study were shown in bold.

**Table 4:** Association of SNPs identified in BC GWAS and risk of BC in present study analysed by Hormone Receptor Status.

SNP ID	Gene Symbol	Risk Allele	EAF^a^	ER+/PR+ (Cases = 408 Control = 1205)			ER−/PR− (Cases = 529 Control = 1205)			TNBC (Cases = 340 Control = 1205)		
				Case/Control	OR^b^ (95% CI)	p-value	Case/Control	OR^b^ (95% CI)	p-value	Case/Control	OR^b^ (95% CI)	p-value
rs1219648	FGFR2	G	0.37	**406/1196**	**1.27 (1.07–1.50)**	**0.005**	526/1196	1.00 (0.85–1.16)	0.989	337/1196	1.03 (0.86–1.23)	0.722
rs2046210	ESR1	T	0.35	407/1198	1.13 (0.96–1.34)	0.132	**527/1198**	**1.21 (1.04–1.41)**	**0.013**	**339/1198**	**1.24 (1.03–1.48)**	**0.019**
rs2981575	FGFR2	T	0.62	**406/1193**	**0.80 (0.67–0.94)**	**0.009**	528/1193	0.97 (0.84–1.13)	0.790	339/1193	0.94 (0.79–1.13)	0.546
rs2981579	FGFR2	T	0.40	**406/1205**	**1.18 (1.007–1.40)**	**0.040**	529/1205	1.01 (0.87–1.17)	0.883	340/1205	1.03 (0.86–1.23)	0.708
rs2981582	FGFR2	T	0.33	**408/1204**	**1.23 (1.04–1.46)**	**0.016**	529/1204	1.07 (0.91–1.25)	0.383	340/1204	1.08 (0.90–1.31)	0.378
rs614367	CCND1	T	0.15	**407/1189**	**1.36 (1.10–1.68)**	**0.004**	520/1189	1.00 (0.81–1.22)	0.978	335/1189	1.12 (0.88–1.41)	0.336
rs704010	ZMIZ1	T	0.29	**407/1198**	**1.21 (1.01–1.44)**	**0.032**	528/1198	0.95 (0.81–1.12)	0.597	340/1198	0.96 (0.79–1.17)	0.744
rs889312	MAP3K1	C	0.39	**404/1178**	**1.26 (1.07–1.49)**	**0.004**	524/1178	1.12 (0.96–1.30)	0.122	337/1178	1.04 (0.88–1.24)	0.601
rs9485372	UST	G	0.79	404/1199	1.06 (0.87–1.29)	0.551	**526/1199**	**1.21 (1.005–1.46)**	**0.044**	**339/1199**	**1.30 (1.04–1.63)**	**0.020**
rs999737	RAD51L1	C	0.88	**408/1204**	**0.78 (0.62–0.99)**	0.044	529/1204	1.04 (0.83–1.31)	0.681	340/1204	1.09 (0.83–1.43)	0.516

Abbreviations: Chr, Chromosome; ESR1, Estrogen receptor 1; CI, Confidence Interval; EAF, Effect Allele Frequency; ER+/PR+, Estrogen Receptor Positive/Progesterone Receptor Positive; ER−/PR−, Estrogen Receptor Negative/Progesterone Receptor Negative; FGFR2, Fibroblast Growth Factor Receptor 2; GWAS, Genome Wide Association Studies; MAP3K1, Mitogen-Activated Kinase Kinase Kinase 1; OR, Odds Ratio; RAD51L1, RAD51 Paralog B; SNP, Single Nucleotide Polymorphism; TNBC, Triple Negative Breast Cancer; UST, Uronyl 2-Sulfotransferase; ZMIZ1, Zinc Finger Miz-Domain Containing 1; ZNF365, Zinc Finger Protein 365.

^a^Effect Allele Frequency in controls.

^b^Adjusted for age and region of residence.

Total number per SNP may vary because of missing values.

Significant association are shown in bold.

**Table 5:** Association between PRS and Breast Cancer risk analysed by menopausal and hormone receptor status.

Parameters	Categories	All Study Participants			Without Family History of Breast, Ovary or Endometrial Cancer		
		N (Case/Control)	OR^a^ (95% CI)	p-value	N (Case/Control)	OR^a^ (95% CI)	p-value
PRS (Total)	≤1.327	266/287	1.00		257/280	1.00	
	1.328–1.620	272/280	1.04 (0.82–1.32)	0.701	264/271	1.06 (0.83–1.34)	0.631
	1.621–1.920	271/281	1.03 (0.82–1.31)	0.748	257/274	1.02 (0.80–1.29)	0.867
	≥1.930	296/256	1.25 (0.98–1.58)	0.063	285/249	1.25 (0.98–1.59)	0.067
	Trend		1.06 (0.99–1.15)	0.081		1.06 (0.98–1.14)	0.102
	Risk per unit increase in PRS		**1.25 (1.03–1.52)**	**0.019**		**1.23 (1.01–1.50)**	**0.032**
PRS (Premenopausal)	≤1.327	153/138	1.00		149/135	1.00	
	1.328–1.620	137/148	0.83 (0.60–1.15)	0.275	135/143	0.85 (0.61–1.19)	0.347
	1.621–1.920	127/160	0.71 (0.51–0.99)	0.045	121/155	0.70 (0.50–0.98)	0.041
	≥1.930	151/151	0.90 (0.65–1.24)	0.531	149/148	0.91 (0.65–1.26)	0.579
	Trend		0.95 (0.86–1.05)	0.392		0.95 (0.86–1.06)	0.400
	Risk per unit increase in PRS		1.01 (0.77–1.31)	0.922		1.01 (0.77–1.32)	0.905
PRS (Postmenopausal)	≤1.327	113/146	1.00		108/142	1.00	
	1.328–1.620	135/130	1.33 (0.95–1.88)	0.101	129/126	1.33 (0.94–1.89)	0.106
	1.621–1.920	143/120	**1.53 (1.08–2.16)**	**0.015**	136/118	**1.50 (1.05–2.13)**	**0.023**
	≥1.930	145/102	**1.83 (1.28–2.60)**	**0.001**	136/98	**1.82 (1.27–2.61)**	**0.001**
	Trend		**1.21 (1.08–1.35)**	**0.001**		**1.21 (1.08–1.35)**	**0.001**
	Risk per unit increase in PRS		**1.60 (1.21–2.13)**	**0.001**		**1.56 (1.16–2.09)**	**0.003**
PRS (ER+/PR+)	≤1.327	89/287	1.00		85/280	1.00	
	1.328–1.620	86/280	0.98 (0.70–1.38)	0.926	83/271	1.00 (0.70–1.41)	0.992
	1.621–1.920	94/281	1.07 (0.76–1.49)	0.680	88/274	1.05 (0.74–1.48)	0.767
	≥1.930	101/256	1.26 (0.90–1.76)	0.167	95/249	1.24 (0.88–1.75)	0.199
	Trend		1.08 (0.97–1.20)	0.142		1.07 (0.96–1.19)	0.192
	Risk per unit increase in PRS		**1.36 (1.04–1.79)**	**0.024**		**1.36 (1.03–1.80)**	**0.030**
PRS (ER−/PR−)	≤1.327	121/287	1.00		117/280	1.00	
	1.328–1.620	136/280	1.15 (0.86–1.55)	0.336	132/271	1.17 (0.86–1.58)	0.303
	1.621–1.920	127/281	1.07 (0.79–1.44)	0.637	120/274	1.05 (0.77–1.42)	0.749
	≥1.930	118/256	1.10 (0.81–1.49)	0.538	115/249	1.11 (0.81–1.51)	0.491
	Trend		1.02 (0.92–1.12)	0.663		1.02 (0.92–1.12)	0.667
	Risk per unit increase in PRS		1.10 (0.86–1.40)	0.441		1.08 (0.84–1.38)	0.521
PRS (TNBC)	≤1.327	81/287	1.00		78/280	1.00	
	1.328–1.620	89/280	1.12 (0.80–1.58)	0.493	86/271	1.14 (0.80–1.61)	0.458
	1.621–1.920	87/281	1.09 (0.77–1.55)	0.592	81/274	1.06 (0.74–1.51)	0.734
	≥1.930	70/256	0.97 (0.67–1.39)	0.872	67/249	0.96 (0.67–1.40)	0.868
	Trend		0.99 (0.88–1.10)	0.874		0.98 (0.87–1.10)	0.803
	Risk per unit increase in PRS		1.04 (0.78–1.39)	0.756		1.01 (0.75–1.35)	0.933

Abbreviations: CI, Confidence Interval; ER+/PR+, Estrogen Receptor Positive/Progesterone Receptor Positive; ER−/PR−, Estrogen Receptor Negative/Progesterone Receptor Negative; OR, Odds Ratio; TNBC, Triple Negative Breast Cancer.

^a^Adjusted for age and region of residence.

Significant associations are shown in bold.
