# Supplementary material for: Association of Genome-Wide Association Study (GWAS) Identified SNPs and Risk of Breast Cancer in an Indian Population
Source: Sci Rep. 2017 Jan 18;7:40963. doi: 10.1038/srep40963 (PMC5241870; doi:10.1038/srep40963)
Supplement: Supplementary Dataset 1 [file srep40963-s1.doc]

**Title:** Association of Genome-Wide Association Study (GWAS) Identified SNPs and Risk of Breast Cancer in An Indian Population

**Authors:** Rajini Nagrani^1^, Sharayu Mhatre^1^, Preetha Rajaraman^2^, Nilanjan Chatterjee^2,3,4^ , Mohammad R Akbari^5,6^, Paolo Boffetta^7^, Paul Brennan^8^, Rajendra Badwe^9^, Sudeep Gupta^9^, Rajesh Dikshit^1^

^1^Centre for Cancer Epidemiology, Tata Memorial Centre, Mumbai, India

^2^Division of Cancer Epidemiology & Genetics, National Cancer Institute, Bethesda, USA

^3^Department of Biostatistics, Bloomberg School of Public Health, Johns Hopkins University, USA

^4^Department of Oncology, School of Medicine, Johns Hopkins University, USA

^5^Women's College Research Institute, Women's College Hospital, Toronto, ON, Canada

^6^Dalla Lana School of Public Health, University of Toronto, Toronto, ON, Canada

^7^Institute For Translational Epidemiology, Mount Sinai Hospital, One Gustave L.Levy Place New York, NY, USA

^8^Genetic Epidemioloy Group, International Agency for Research on Cancer, 150 Cours Albert Thomas, 69372 Lyon CEDEX, France

^9^Department of Surgical Oncology, Tata Memorial Hospital, Mumbai, India

Corresponding Author: Dr. Rajesh Dikshit

Affiliation: Centre for Cancer Epidemiology, Tata Memorial Centre, E.Borges Road, Parel Mumbai, Maharashtra, India. Pin 400 012.

Phone: +91 9969518844

E-mail Address: [**dixr24@hotmail.com**](mailto:dixr24@hotmail.com)

| **Supplementary Table S1: Association of SNPs identified in BC GWAS and risk of BC in present study stratified on Menopausal Status** | | | | | | | | | |
| --- | --- | --- | --- | --- | --- | --- | --- | --- | --- |
| **SNP ID** | **Gene Symbol** | **Effect Allele** | **EAF^a^** | **Postmenopausal**  **(Cases=586 Control= 541)** | | | **Premenopausal**  **(Cases=607 Control= 650)** | | |
|  |  |  |  | **Case/Control** | **OR^b^ (95% CI)** | **p-value** | **Case/Control** | **OR^b^ (95% CI)** | **p-value** |
| rs10069690 | TERT | T | 0.29 | 581/534 | 0.96 (0.80-1.15) | 0.684 | 605/649 | 0.92 (0.77-1.10) | 0.404 |
| rs1011970 | CDKN2BAS | T | 0.26 | 582/540 | 1.12 (0.93-1.36) | 0.223 | 604/647 | 1.14 (0.95-1.37) | 0.132 |
| rs10822013 | ZNF365 | T | 0.50 | 584/539 | 0.89 (0.75-1.05) | 0.184 | 603/640 | 1.12 (0.95-1.32) | 0.167 |
| rs10941679 | FGF10 | G | 0.39 | 583/539 | 0.96 (0.82-1.14) | 0.718 | 606/647 | 1.04 (0.89-1.23) | 0.579 |
| rs10995190 | ZNF365 | G | 0.92 | 586/541 | 1.34 (0.98-1.82) | 0.062 | 607/650 | 0.94 (0.69-1.27) | 0.704 |
| rs11249433 | LOC647121 | C | 0.17 | 575/534 | 1.02 (0.81-1.28) | 0.846 | 598/642 | 1.06 (0.86-1.31) | 0.559 |
| rs1219648 | FGFR2 | G | 0.37 | **584/537** | **1.22 (1.02-1.45)** | **0.026** | 603/645 | 1.04 (0.88-1.22) | 0.632 |
| rs13281615 | FAM84B | G | 0.50 | 579/533 | 1.12(0.94-1.32) | 0.179 | 603/642 | 1.01 (0.86-1.18) | 0.874 |
| rs13387042 | TNP1 | A | 0.52 | 583/539 | 0.92 (0.78-1.09) | 0.360 | 604/643 | 0.93 (0.79-1.09) | 0.410 |
| rs1562430 | FAM84B | A | 0.77 | 583/541 | 0.97 (0.79-1.18) | 0.771 | 601/649 | 1.02 (0.84-1.24) | 0.795 |
| rs2046210 | ESR1 | T | 0.35 | **584/539** | **1.22 (1.01-1.46)** | **0.031** | 604/645 | 1.09 (0.93-1.28) | 0.263 |
| rs2180341 | RNF146 | G | 0.41 | 580/536 | 1.01 (0.86-1.20) | 0.834 | 601/642 | 0.89 (0.75-1.04) | 0.156 |
| rs2981575 | FGFR2 | T | 0.62 | **582/538** | **0.81 (0.68-0.96)** | **0.019** | 604/641 | 0.95 (0.81-1.12) | 0.585 |
| rs2981579 | FGFR2 | T | 0.40 | **585/541** | **1.19 (1.00-1.42)** | **0.040** | 606/650 | 1.03 (0.88-1.21) | 0.680 |
| rs2981582 | FGFR2 | T | 0.33 | **586/541** | **1.29 (1.07-1.55)** | **0.006** | 607/649 | 1.05 (0.88-1.24) | 0.557 |
| rs3112612 | TOX3 | T | 0.46 | 582/539 | 1.00 (0.85-1.18) | 0.927 | 604/644 | 1.07 (0.91-1.24) | 0.395 |
| rs3757318 | C6orf97 | A | 0.07 | 585/539 | 1.06 (0.75-1.49) | 0.739 | 607/649 | 0.93 (0.69-1.25) | 0.654 |
| rs3803662 | TOX3 | T | 0.28 | 583/535 | 1.09 (0.91-1.31) | 0.333 | 606/646 | 1.07 (0.90-1.28) | 0.407 |
| rs3817198 | LSP1 | C | 0.36 | 582/537 | 1.07 (0.90-1.28) | 0.393 | 606/643 | 1.04 (0.89-1.22) | 0.583 |
| rs4415084 | FGF10 | T | 0.52 | 581/539 | 0.98 (0.82-1.16) | 0.839 | 602/644 | 1.08 (0.92-1.27) | 0.317 |
| rs4784227 | TOX3 | T | 0.22 | 584/540 | 1.08 (0.89-1.31) | 0.412 | 604/647 | 1.12 (0.93-1.35) | 0.225 |
| rs4973768 | SLC4A7 | T | 0.45 | 584/538 | 1.02 (0.86-1.21) | 0.754 | 604/646 | 1.03 (0.88-1.21) | 0.650 |
| rs614367 | CCND1 | T | 0.15 | 578/540 | 1.23 (0.97-1.54) | 0.077 | 604/635 | 1.06 (0.85-1.32) | 0.579 |
| rs6504950 | STXBP4 | G | 0.84 | 585/540 | 0.97 (0.77-1.22) | 0.830 | 604/649 | 0.87 (0.70-1.08) | 0.218 |
| rs704010 | ZMIZ1 | A | 0.29 | 585/536 | 1.08 (0.90-1.30) | 0.383 | 606/648 | 1.05 (0.88-1.25) | 0.552 |
| rs8170 | C19orf62 | A | 0.10 | 583/541 | 1.22 (0.93-1.61) | 0.137 | 606/649 | 0.89 (0.69-1.16) | 0.427 |
| rs865686 | 9q31.2 | T | 0.86 | **584/539** | **1.30 (1.02-1.65)** | **0.030** | 606/646 | 1.21 (0.96-1.54) | 0.102 |
| rs889312 | MAP3K1 | C | 0.39 | **578/529** | **1.24 (1.04-1.47)** | **0.013** | 599/635 | 1.10 (0.94-1.30) | 0.210 |
| rs9485372 | UST | G | 0.79 | 578/537 | 1.07 (0.87-1.31) | 0.509 | 604/648 | 1.12 (0.92-1.37) | 0.250 |
| rs999737 | RAD51L1 | C | 0.88 | 586/540 | 1.13 (0.88-1.46) | 0.316 | **607/650** | **0.78 (0.61-0.99)** | **0.045** |
| Abbreviations: C19orf62, Chromosome 19 Open Reading Frame 62; C6orf97, Chromosome 6 Open Reading Frame 97; CCND1, Cyclin D1; CDKN2BAS, Cyclin-Dependent Kinase Inhibitor 2B, Antisense; Chr, Chromosome; CI, Confidence interval; RAF, Effect Allele Frequency; ESR1, Estrogen receptor 1; FAM48B, Family With Sequence Similarity 84, Member B; FGF10, Fibroblast Growth Factor 10; FGFR2, Fibroblast Growth Factor Receptor 2; GWAS, Genome Wide Association Studies; HCN1, Hyperpolarization-Activated Cyclic Nucleotide-Gated Potassium Channel 1; LSP1, Lymphocyte-Specific Protein; MAP3K1, Mitogen-Activated Kinase Kinase Kinase 1; OR, Odds Ratio; RAD51L1, RAD51 Paralog B; RNF146, Ring Finger Protein 146; SNP, Single Nucleotide Polymorphism; STXBP4, Syntaxin-Binding Protein 4; TERT, Telomerase Reverse Transcriptase; TNP1, Transition Protein 1; TOX3, Tox High Mobility Group Box Family Member 3; UST, Uronyl 2-Sulfotransferase;, ZMIZ1, Zinc Finger Miz-Domain Containing 1; ZNF365, Zinc Finger Protein 365. ^a^Effect Allele Frequency in controls.  ^b^Adjusted for age and region of residence. Total number per SNP may vary because of missing values. Significant association are shown in bold. | | | | | | | | | |

| **Supplementary Table S2: Association of SNPs identified in BC GWAS and risk of BC in present study analyzed by Hormone Receptor Status** | | | | | | | | | | | | | | | | | | | | | | | | |
| --- | --- | --- | --- | --- | --- | --- | --- | --- | --- | --- | --- | --- | --- | --- | --- | --- | --- | --- | --- | --- | --- | --- | --- | --- |
| **SNP ID** | | **Gene Symbol** | | | **Effect Allele** | **EAF^a^** | **ER+/PR+**  **(Cases=408 Control= 1205)** | | | | | | **ER-/PR-**  **(Cases=529 Control= 1205)** | | | | | | **TNBC**  **(Cases=340 Control= 1205)** | | | | | |
|  |  |  |  |  |  |  | **Case/**  **Control** | | **OR_ER+/PR+_^b^**  **(95% CI)** | | | **p-value** | **Case/**  **Control** | **OR_ER-/PR-_^b^**  **(95% CI)** | | | **p-value** | | **Case/**  **Control** | | **OR_TNBC_^b^**  **(95% CI)** | | **p-value** | |
| rs10069690 | | TERT | | | T | 0.29 | 405/1197 | | 0.86 (0.72-1.03) | | | 0.118 | 526/1197 | 1.02 (0.87-1.19) | | | 0.794 | | 338/1197 | | 1.02 (0.84-1.22) | | 0.834 | |
| rs1011970 | | CDKN2BAS | | | T | 0.26 | 406/1201 | | 1.16 (0.97-1.39) | | | 0.096 | 525/1201 | 1.04 (0.88-1.23) | | | 0.576 | | 339/1201 | | 1.04 (0.88-1.26) | | 0.684 | |
| rs10822013 | | ZNF365 | | | T | 0.50 | 406/1193 | | 1.13 (0.95-1.33) | | | 0.143 | 527/1193 | 0.95 (0.82-1.11) | | | 0.572 | | 339/1193 | | 0.95 (0.80-1.13) | | 0.617 | |
| rs10941679 | | FGF10 | | | G | 0.39 | 408/1200 | | 1.01 (0.86-1.19) | | | 0.847 | 527/1200 | 0.98 (0.84-1.14) | | | 0.843 | | 338/1200 | | 0.95 (0.80-1.14) | | 0.615 | |
| rs10995190 | | ZNF365 | | | G | 0.92 | 408/1205 | | 0.96 (0.72-1.29) | | | 0.831 | 529/1205 | 1.24 (0.93-1.65) | | | 0.130 | | 340/1205 | | 1.21 (0.86-1.69) | | 0.261 | |
| rs11249433 | | LOC647121 | | | G | 0.17 | 399/1190 | | 1.19 (0.96-1.48) | | | 0.094 | 525/1190 | 0.93 (0.76-1.14) | | | 0.500 | | 337/1190 | | 0.96 (0.75-1.21) | | 0.735 | |
| rs1219648 | | FGFR2 | | | G | 0.37 | **406/1196** | | **1.27 (1.07-1.50)** | | | **0.005** | 526/1196 | 1.00 (0.85-1.16) | | | 0.989 | | 337/1196 | | 1.03 (0.86-1.23) | | 0.722 | |
| rs13281615 | | FAM84B | | | G | 0.50 | 401/1189 | | 1.02 (0.87-1.20) | | | 0.730 | 528/1189 | 1.08 (0.94-1.25) | | | 0.256 | | 339/1189 | | 1.08 (0.91-1.28) | | 0.369 | |
| rs13387042 | | TNP1 | | | A | 0.52 | 405/1196 | | 0.98 (0.83-1.15) | | | 0.860 | 527/1196 | 0.89 (0.77-1.03) | | | 0.140 | | 338/1196 | | 0.88 (0.74-1.05) | | 0.164 | |
| rs1562430 | | FAM84B | | | A | 0.77 | 404/1204 | | 1.02 (0.84-1.23) | | | 0.826 | 529/1204 | 0.88 (0.75-1.05) | | | 0.171 | | 340/1204 | | 0.91 (0.74-1.11) | | 0.370 | |
| rs2046210 | | ESR1 | | | T | 0.35 | 407/1198 | | 1.13 (0.96-1.34) | | | 0.132 | **527/1198** | **1.21 (1.04-1.41)** | | | **0.013** | | **339/1198** | | **1.24 (1.03-1.48)** | | **0.019** | |
| rs2180341 | | RNF146 | | | G | 0.41 | 402/1192 | | 0.92 (0.78-1.09) | | | 0.362 | 526/1192 | 0.94 (0.82-1.09) | | | 0.477 | | 339/1192 | | 0.91 (0.77-1.08) | | 0.329 | |
| rs2981575 | | FGFR2 | | | T | 0.62 | **406/1193** | | **0.80 (0.67-0.94)** | | | **0.009** | 528/1193 | 0.97 (0.84-1.13) | | | 0.790 | | 339/1193 | | 0.94 (0.79-1.13) | | 0.546 | |
| rs2981579 | | FGFR2 | | | T | 0.40 | **406/1205** | | **1.18 (1.007-1.40)** | | | **0.040** | 529/1205 | 1.01 (0.87-1.17) | | | 0.883 | | 340/1205 | | 1.03 (0.86-1.23) | | 0.708 | |
| rs2981582 | | FGFR2 | | | T | 0.33 | **408/1204** | | **1.23 (1.04-1.46)** | | | **0.016** | 529/1204 | 1.07 (0.91-1.25) | | | 0.383 | | 340/1204 | | 1.08 (0.90-1.31) | | 0.378 | |
| rs3112612 | | TOX3 | | | T | 0.46 | 404/1197 | | 1.02 (0.87-1.20) | | | 0.733 | 527/1197 | 1.00 (0.85-1.16) | | | 0.939 | | 339/1197 | | 0.97 (0.82-1.15) | | 0.778 | |
| rs3757318 | | C6orf97 | | | A | 0.07 | 408/1202 | | 1.00 (0.73-1.38) | | | 0.954 | 528/1202 | 0.95 (0.71-1.26) | | | 0.729 | | 340/1202 | | 0.74 (0.51-1.08) | | 0.128 | |
| rs3803662 | | TOX3 | | | A | 0.28 | 407/1195 | | 1.15 (0.96-1.37) | | | 0.117 | 527/1195 | 1.04 (0.88-1.22) | | | 0.613 | | 339/1195 | | 0.98 (0.81-1.18) | | 0.836 | |
| rs3817198 | | LSP1 | | | C | 0.36 | 404/1194 | | 1.11 (0.94-1.31) | | | 0.179 | 529/1194 | 0.95 (0.82-1.10) | | | 0.544 | | 340/1194 | | 0.96 (0.80-1.14) | | 0.689 | |
| rs4415084 | | FGF10 | | | T | 0.52 | 406/1197 | | 1.15 (0.97-1.35) | | | 0.093 | 525/1197 | 0.93 (0.80-1.09) | | | 0.410 | | 339/1197 | | 0.95 (0.80-1.14) | | 0.624 | |
| rs4784227 | | TOX3 | | | T | 0.22 | 407/1201 | | 1.17 (0.97-1.41) | | | 0.085 | 527/1201 | 1.04 (0.87-1.23) | | | 0.644 | | 339/1201 | | 0.95 (0.77-1.17) | | 0.637 | |
| rs4973768 | | SLC4A7 | | | T | 0.45 | 404/1198 | | 1.06 (0.90-1.25) | | | 0.441 | 529/1198 | 1.02 (0.88-1.18) | | | 0.723 | | 340/1198 | | 0.95 (0.80-1.13) | | 0.601 | |
| rs614367 | | CCND1 | | | T | 0.15 | **407/1189** | | **1.36 (1.10-1.68)** | | | **0.004** | 520/1189 | 1.00 (0.81-1.22) | | | 0.978 | | 335/1189 | | 1.12 (0.88-1.41) | | 0.336 | |
| rs6504950 | | STXBP4 | | | G | 0.84 | 406/1203 | | 0.98 (0.79-1.22) | | | 0.915 | 529/1203 | 0.94 (0.77-1.15) | | | 0.608 | | 340/1203 | | 0.89 (0.70-1.12) | | 0.331 | |
| rs704010 | | ZMIZ1 | | | T | 0.29 | **407/1198** | | **1.21 (1.01-1.44)** | | | **0.032** | 528/1198 | 0.95 (0.81-1.12) | | | 0.597 | | 340/1198 | | 0.96 (0.79-1.17) | | 0.744 | |
| rs8170 | | C19orf62 | | | A | 0.10 | 406/1204 | | 0.95 (0.72-1.24) | | | 0.709 | 528/1204 | 1.10 (0.87-1.38) | | | 0.420 | | 340/1204 | | 1.24 (0.95-1.61) | | 0.101 | |
| rs865686 | | 9q31.2 | | | T | 0.86 | 407/1199 | | 1.23 (0.97-1.56) | | | 0.081 | 528/1199 | 1.17 (0.94-1.45) | | | 0.139 | | 339/1199 | | 1.25 (0.96-1.62) | | 0.089 | |
| rs889312 | | MAP3K1 | | | C | 0.39 | **404/1178** | | **1.26 (1.07-1.49)** | | | **0.004** | 524/1178 | 1.12 (0.96-1.30) | | | 0.122 | | 337/1178 | | 1.04 (0.88-1.24) | | 0.601 | |
| rs9485372 | | UST | | | G | 0.79 | 404/1199 | | 1.06 (0.87-1.29) | | | 0.551 | **526/1199** | **1.21 (1.005-1.46)** | | | **0.044** | | **339/1199** | | **1.30 (1.04-1.63)** | | **0.020** | |
| rs999737 | | RAD51L1 | | | C | 0.88 | **408/1204** | | **0.78 (0.62-0.99)** | | | **0.044** | 529/1204 | 1.04 (0.83-1.31) | | | 0.681 | | 340/1204 | | 1.09 (0.83-1.43) | | 0.516 | |
| Abbreviations: C19orf62, Chromosome 19 Open Reading Frame 62; C6orf97, Chromosome 6 Open Reading Frame 97; CCND1, Cyclin D1; CDKN2BAS, Cyclin-Dependent Kinase Inhibitor 2B, Antisense; Chr, Chromosome; ESR1, Estrogen receptor 1; CI, Confidence Interval; EAF, Effect Allele Frequency; ER+/PR+, Estrogen Receptor Positive/ Progesterone Receptor Positive; ER-/PR-, Estrogen Receptor Negative/ Progesterone Receptor Negative; FAM48B, Family With Sequence Similarity 84, Member B; FGF10, Fibroblast Growth Factor 10; FGFR2, Fibroblast Growth Factor Receptor 2; GWAS, Genome Wide Association Studies; HCN1, Hyperpolarization-Activated Cyclic Nucleotide-Gated Potassium Channel 1; LSP1, Lymphocyte-Specific Protein; MAP3K1, Mitogen-Activated Kinase Kinase Kinase 1; OR, Odds Ratio; RAD51L1, RAD51 Paralog B; RNF146, Ring Finger Protein 146; SNP, Single Nucleotide Polymorphism; STXBP4, Syntaxin-Binding Protein 4; TERT, Telomerase Reverse Transcriptase; TNBC, Triple Negative Breast Cancer; TNP1, Transition Protein 1; TOX3, Tox High Mobility Group Box Family Member 3; UST, Uronyl 2-Sulfotransferase;, ZMIZ1, Zinc Finger Miz-Domain Containing 1; ZNF365, Zinc Finger Protein 365. ^a^Effect Allele Frequency in controls. ^b^Adjusted for age and region of residence. Total number per SNP may vary because of missing values. Significant associations are shown in bold | | | | | | | | | | | | | | | | | | | | | | | | |
|  | **Supplementary Table S3: Comparison of BC SNPs identified previously in candidate studies with Indian population** | | | | | | | | | | | | | | | | | | | | | | |  |
|  | **Variant** | | **Chr** | **Nearby Gene** | | **Position** | | **Previous Candidate SNP studies** | | | | | | | **Present Case-Control Study (Cases=1,194 Control= 1,205)** | | | | | | | | |  |
|  |  |  |  |  |  |  |  | **OR** | | **95%CI** | **p-Value** | | **PubMed ID** | | **Minor Allele** | **MAF^a^** | | **Ca/Co** | | **OR^b^ (95% CI)** | | **p-value** | |  |
|  | rs2420946 | | 10 | FGFR2 | | 123341314 | | 1.52 | | 1.36-1.69 | NR | | 20300826 | | T | 0.38 | | **1187/1189** | | **1.16 (1.03-1.31)** | | **0.011** | |  |
|  | rs3218408 | | 7 | XRCC2 | | 151805264 | | 1.33 | | 1.12-1.57 | NR | | 21632523 | | G | 0.19 | | **1181/1192** | | **1.16 (1.01-1.34)** | | **0.035** | |  |
|  | rs1641535 | | 17 | ATP1B2 | | 7486858 | | 0.88 | | 0.77-0.99 | 0.001 | | 17683073 | | A | 0.21 | | **1187/1188** | | **1.15 (1.007-1.32)** | | **0.039** | |  |
|  | rs1641536 | | 17 | ATP1B2 | | 7486709 | | 0.88 | | 0.77-0.99 | 0.0005 | | 17683073 | | A | 0.21 | | **1189/1192** | | **1.15 (1.004-1.32)** | | **0.043** | |  |
|  | rs861539 | | 14 | XRCC3 | | 103235506 | | 1.16 | | 1.04-1.30 | 0.009 | | 16485136 | | T | 0.18 | | 1191/1179 | | 1.15 (0.99-1.33) | | 0.051 | |  |
|  | rs4919687 | | 10 | CYP17A1 | | 104585238 | | 1.17 | | 1.03-1.34 | 0.03 | | 18006912 | | A | 0.11 | | 1190/1195 | | 0.84 (0.69-1.01) | | 0.077 | |  |
|  | rs2070744 | | 7 | NOS3 | | 150127727 | | 0.6 | | 0.42-0.86 | NR | | 20204503 | | C | 0.22 | | 1191/1199 | | 1.13 (0.98-1.29) | | 0.078 | |  |
|  | rs231775 | | 2 | CTLA4 | | 204558220 | | 1.31 | | 1.17-1.48 | <0.00001 | | 20920330 | | G | 0.34 | | 1188/1196 | | 0.89 (0.79-1.01) | | 0.088 | |  |
|  | rs4919682 | | 10 | CYP17A1 | | 104574320 | | 1.16 | | 1.01-1.33 | 0.04 | | 18006912 | | T | 0.08 | | 1194/1198 | | 0.83 (0.67-1.03) | | 0.106 | |  |
|  | rs1136201 | | 17 | ERBB2 | |  | | 1.28 | | 1.04-1.58 | 0.0225 | | 18438707 | | G | 0.13 | | 1190/1197 | | 1.12 (0.95-1.33) | | 0.156 | |  |
|  | rs1799794 | | 14 | XRCC3 | | 103249020 | | 1.11 | | 1.03-1.19 | NR | | 20049524 | | G | 0.42 | | 1189/1192 | | 0.92 (0.82-1.03) | | 0.177 | |  |
|  | rs1801133 | | 1 | MTHFR | | 11790644 | | 0.935 | | 0.887-0.986 | 0.013 | | 23217001 | | T | 0.15 | | 1187/1195 | | 0.89 (0.76-1.05) | | 0.182 | |  |
|  | rs1805794 | | 8 | NBN | | 91059655 | | 1.06 | | 0.97-1.16 | 0.18 | | 19393077 | | G | 0.43 | | 1191/1197 | | 0.92 (0.82-1.03) | | 0.184 | |  |
|  | rs1695 | | 11 | GSTP1 | | 67109265 | | 2.15 | | 1.30-3.56 | NR | | 11950848 | | G | 0.28 | | 1189/1190 | | 0.91 (0.80-1.04) | | 0.185 | |  |
|  | rs1800067 | | 16 | ERCC4 | | 13936534 | | 2.34 | | 1.17-4.69 | 0.017 | | 19116388 | | A | 0.03 | | 1193/1200 | | 0.80 (0.58-1.11) | | 0.185 | |  |
|  | rs1056836 | | 2 | CYP1B1 | | 38209854 | | 1.09 | | 0.79-1.50 | NR | | 11950848 | | G | 0.2 | | 1186/1192 | | 1.08 (0.94-1.24) | | 0.242 | |  |
|  | rs1045485 | | 2 | CASP8 | | 201975095 | | 0.73 | | 0.60-0.90 | 5.7 10^–7^ | | 17293864 | | C | 0.04 | | 1194/1200 | | 1.18 (0.89-1.57) | | 0.243 | |  |
|  | rs4986938 | | 14 | ESR2 | | 63769569 | | 0.944 | | 0.895-0.997 | 0.037 | | 20390341 | | A | 0.28 | | 1188/1196 | | 0.93 (0.82-1.05) | | 0.277 | |  |
|  | rs1801516 | | 11 | ATM | | 107680672 | | 2.15 | | 1.37-3.38 | NR | | 20799949 | | A | 0.06 | | 1191/1196 | | 1.12 (0.89-1.41) | | 0.303 | |  |
|  | rs2234767 | | 10 | FAS | | 90739236 | | 1.18 | | 1.04-1.35 | NR | | 19168581 | | A | 0.22 | | 1190/1199 | | 0.93 (0.81-1.07) | | 0.349 | |  |
|  | rs1800629 | | 6 | TNF | | 31651010 | | 1.1 | | 1.04-1.17 | NR | | 20035378 | | A | 0.05 | | 1186/1196 | | 1.11 (0.87-1.43) | | 0.379 | |  |
|  | rs2854744 | | 7 | IGFBP3 | | 45734315 | | 0.92 | | 0.86-0.99 | NR | | 17293864 | | A | 0.5 | | 1190/1197 | | 0.94 (0.84-1.06) | | 0.387 | |  |
|  | rs5186 | | 3 | AGTR1 | | 149942686 | | 0.5 | | 0.23-1.06 | NR | | 21638051 | | C | 0.06 | | 1194/1199 | | 1.10 (0.87-1.40) | | 0.390 | |  |
|  | rs11568785 | | 9 | TGFBR1 | | 98985389 | | 1.16 | | 1.01-1.34 | 0.04 | | 19882361 | | G | 0.03 | | 1194/1198 | | 0.88 (0.62-1.23) | | 0.467 | |  |
|  | rs1799983 | | 7 | NOS3 | | 150133759 | | 0.78 | | 0.61-0.98 | NR | | 20204503 | | T | 0.18 | | 1187/1184 | | 1.05 (0.90-1.22) | | 0.517 | |  |
|  | rs6964587 | | 7 | AKAP9 | | 91275271 | | 1.17 | | 1.08-1.27 | 0.0003 | | 18334708 | | T | 0.38 | | 1190/1198 | | 1.03 (0.92-1.16) | | 0.531 | |  |
|  | rs1800566 | | 16 | NQO1 | | 68302646 | | 1.15 | | 1.01-1.31 | 0.03 | | 20526805 | | T | 0.33 | | 1189/1196 | | 1.03 (0.92-1.17) | | 0.540 | |  |
|  | rs1801270 | | 6 | CDKN1A | | 36759949 | | 1.51 | | 1.17-1.93 | NR | | 21415438 | | A | 0.13 | | 1193/1193 | | 1.05 (0.88-1.24) | | 0.577 | |  |
|  | rs2287499 | | 17 | WRAP53 | | 7532893 | | 1.6 | | 1.04-2.47 | 0.01 | | 17683073 | | G | 0.26 | | 1188/1192 | | 0.96 (0.84-1.09) | | 0.591 | |  |
|  | rs1042838 | | 11 | PGR | | 100438622 | | 1.17 | | 0.93-1.47 | 0.17 | | 16614108 | | T | 0.06 | | 1187/1199 | | 1.05 (0.84-1.32) | | 0.625 | |  |
|  | rs11614913 | | 12 | pre-miRNA | | 52671866 | | 1.3 | | 1.01-1.68 | NR | | 20640596 | | T | 0.27 | | 1193/1198 | | 1.03 (0.90-1.17) | | 0.652 | |  |
|  | rs17468277 | | 2 | CASP8 | | 201979706 | | 0.96 | | 0.92-0.99 | NR | | 21194473 | | T | 0.04 | | 1194/1199 | | 1.06 (0.79-1.43) | | 0.653 | |  |
|  | rs2273535 | | 20 | AURKA | | 54394948 | | 1.35 | | 1.12-1.64 | 0.002 | | 15802297 | | T | 0.31 | | 1190/1192 | | 1.02 (0.90-1.16) | | 0.662 | |  |
|  | rs5275 | | 1 | PTGS2 | | 183374715 | | 0.8 | | 0.66-0.97 | 0.02 | | 17214885 | | C | 0.38 | | 1168/1178 | | 1.02 (0.91-1.15) | | 0.664 | |  |
|  | rs570613 | | 10 | GATA3 | | 8146508 | | 0.82 | | 0.69-0.96 | 0.004 | | 18006915 | | G | 0.23 | | 1186/1190 | | 0.97 (0.84-1.11) | | 0.688 | |  |
|  | rs4680 | | 22 | COMT | | 18325825 | | 1.14 | | 1.03-1.26 | 0.01 | | 18194538 | | A | 0.42 | | 1191/1199 | | 1.02 (0.91-1.14) | | 0.708 | |  |
|  | rs1801320 | | 15 | RAD51 | | 38774820 | | 0.995 | | 0.991-0.998 | NR | | 20454923 | | C | 0.13 | | 1188/1190 | | 0.97 (0.81-1.15) | | 0.745 | |  |
|  | rs1625895 | | 17 | TP53 | | 7518840 | | 0.3 | | 0.15-0.57 | NR | | 11950848 | | A | 0.18 | | 1192/1196 | | 1.02 (0.88-1.18) | | 0.766 | |  |
|  | rs1042522 | | 17 | TP53 | | 7520197 | | 0.7 | | 0.51-0.95 | NR | | 11950848 | | G | 0.48 | | 1190/1195 | | 0.98 (0.87-1.10) | | 0.801 | |  |
|  | rs1799796 | | 14 | XRCC3 | | 103235680 | | 0.21 | | 0.77-0.96 | NR | | 20049524 | | G | 0.19 | | 1186/1191 | | 1.00 (0.87-1.16) | | 0.900 | |  |
|  | rs1801157 | | 10 | CXCL12 | | 44188263 | | 1.44 | | 1.21-1.72 | <0.001 | | 21643956 | | A | 0.22 | | 1191/1196 | | 0.99 (0.86-1.13) | | 0.926 | |  |
|  | rs10262966 | | 7 | POR | | 75227976 | | 1.58 | | 1.04-2.41 | 0.03 | | 17440066 | | G | 0.06 | | 1189/1193 | | 1.00 (0.79-1.26) | | 0.980 | |  |
|  | Abbreviations: Chr, Chromosome; CI, Confidence Interval; MAF, Minor Allele Frequency; OR, Odds Ratio; SNP, Single Nucleotide Polymorphism. ^a^Minor Allele Frequency in controls. ^b^Adjusted on age and region of residence. Odds Ratio fitted for Additive model. Significant associations are shown in bold | | | | | | | | | | | | | | | | | | | | | | |  |

| **Supplementary Table S4: Power of Replication of previously BC GWAS-identified SNPs** | | | | | | |
| --- | --- | --- | --- | --- | --- | --- |
| **SNP ID** | **Power (All)** | **Power (Postmenopausal)** | **Power (Premenopausal)** | **Power (ER+/PR)** | **Power (ER-/PR-)** | **Power (TNBC)** |
| rs10069690 | 0.347 | 0.195 | 0.201 | 0.207 | 0.235 | 0.235 |
| rs1011970 | 0.153 | 0.1 | 0.102 | 0.107 | 0.115 | 0.115 |
| rs10822013 | 0.281 | 0.162 | 0.166 | 0.167 | 0.19 | 0.19 |
| rs10941679 | 0.31 | 0.176 | 0.181 | 0.185 | 0.209 | 0.209 |
| rs10995190 | 0.159 | 0.102 | 0.104 | 0.095 | 0.108 | 0.108 |
| rs11249433 | 0.125 | 0.086 | 0.088 | 0.092 | 0.099 | 0.099 |
| rs1219648 | 0.897 | 0.618 | 0.632 | 0.64 | 0.713 | 0.713 |
| rs13281615 | 0.182 | 0.113 | 0.116 | 0.116 | 0.13 | 0.13 |
| rs13387042 | 0.357 | 0.2 | 0.206 | 0.206 | 0.237 | 0.237 |
| rs1562430 | 0.319 | 0.181 | 0.185 | 0.178 | 0.208 | 0.208 |
| rs2046210 | 0.375 | 0.21 | 0.215 | 0.221 | 0.252 | 0.252 |
| rs2180341 | 0.986 | 0.829 | 0.842 | 0.844 | 0.902 | 0.902 |
| rs2981575 | 0.818 | 0.519 | 0.534 | 0.531 | 0.607 | 0.607 |
| rs2981579 | 0.823 | 0.524 | 0.566 | 0.544 | 0.618 | 0.618 |
| rs2981582 | 0.771 | 0.473 | 0.486 | 0.496 | 0.564 | 0.564 |
| rs3112612 | 0.398 | 0.221 | 0.228 | 0.229 | 0.265 | 0.265 |
| rs3757318 | 0.159 | 0.102 | 0.104 | 0.115 | 0.123 | 0.123 |
| rs3803662 | 0.567 | 0.392 | 0.404 | 0.416 | 0.474 | 0.474 |
| rs3817198 | 0.125 | 0.086 | 0.088 | 0.089 | 0.096 | 0.096 |
| rs4415084 | 0.477 | 0.266 | 0.274 | 0.275 | 0.317 | 0.317 |
| rs4784227 | 0.608 | 0.347 | 0.357 | 0.372 | 0.424 | 0.424 |
| rs4973768 | 0.212 | 0.128 | 0.131 | 0.132 | 0.148 | 0.148 |
| rs614367 | 0.401 | 0.223 | 0.231 | 0.246 | 0.275 | 0.275 |
| rs6504950 | 0.081 | 0.065 | 0.066 | 0.063 | 0.072 | 0.072 |
| rs704010 | 0.138 | 0.092 | 0.094 | 0.097 | 0.105 | 0.105 |
| rs8170 | 0.183 | 0.114 | 0.116 | 0.126 | 0.137 | 0.137 |
| rs865686 | 0.156 | 0.101 | 0.103 | 0.097 | 0.124 | 0.124 |
| rs889312 | 0.271 | 0.157 | 0.161 | 0.164 | 0.186 | 0.186 |
| rs9485372 | 0.174 | 0.11 | 0.112 | 0.108 | 0.134 | 0.134 |
| rs999737 | 0.103 | 0.076 | 0.077 | 0.073 | 0.088 | 0.088 |
| Abbreviations: BC, Breast Cancer; ER+/PR+, Estrogen Receptor Positive/ Progesterone Receptor Positive; ER-/PR-, Estrogen Receptor Negative/ Progesterone Receptor Negative; GWAS, Genome Wide Association Studies; TNBC, Triple Negative Breast Cancer | | | | | | |
